# Morphological Root Responses and Molecular Regulation of Cation Transporters Are Differently Affected by Copper Toxicity and Cropping System Depending on the Grapevine Rootstock Genotype

**DOI:** 10.3389/fpls.2019.00946

**Published:** 2019-07-19

**Authors:** Laura Marastoni, Michele Sandri, Youry Pii, Fabio Valentinuzzi, Stefano Cesco, Tanja Mimmo

**Affiliations:** Faculty of Science and Technology, Free University of Bozen-Bolzano, Bolzano, Italy

**Keywords:** grapevine, oat, copper, intercropping, gene expression, bivalent cations transporters

## Abstract

The high copper (Cu) concentration in vineyard soils causes the increase of Cu toxicity symptoms in young grapevines. Recently, intercropping of grapevine and oat was shown to reduce Cu toxicity effects, modulating the root ionome. On these bases, the focus of the work was to investigate the impact of Cu toxicity of either monocropped or oat-intercropped grapevine rootstocks plants (196.17 and Fercal), at both phenotypic (i.e., root architecture), and molecular (i.e., expression of transporters) levels. The results showed a different response in terms of root morphology that are both rootstock- and cropping system dependent. Moreover, the expression pattern of transporter genes (i.e., *VvCTr*, *VvNRAMP*, and *VvIRT1*) in monocropped grapevine might resemble a Mn deficiency response induced by the excess of Cu, especially in Fercal plants. The gene expression in intercropped grapevines suggested rootstock-specific response mechanisms, depending on Cu levels. In fact, at low Cu concentrations, Fercal enhanced both root system growth and transporter genes expression; contrarily, 196.17 increased apoplast divalent cations accumulation and transporters expression. At high Cu concentrations, Fercal increased the expression of all bivalent cation transporters and, as previously observed, enhanced the release of root exudates, whereas the 196.17 only modulated transporters. In conclusion, our results might suggest that the different adaptation strategies of the two rootstocks to Cu toxicity could be mainly ascribable to a fine-tuning of bivalent cations transporters expression at root level.

## Introduction

The continuous application of copper (Cu)-based fungicides in viticulture is inducing an increase of Cu concentration in vineyard soils worldwide (Mackie et al., 2012). A survey carried out on vineyard soil on different European soil samples showed that Cu levels might be extremely variable, ranging from 350 to 690 mg Cu kg^−1^, largely exceeding the limits imposed (Ruyters et al., 2013). Indeed, vineyard soil Cu contamination has already been reported in Italy, south France, and Germany (Deluisa et al., 1996; Ribolzi et al., 2002; Provenzano et al., 2010; El Azzi et al., 2013; Steinmetz et al., 2017). Grapevine plants, especially the young rootstocks featuring a shallow root apparatus, have to cope with the increasing metal concentration in soils and often exhibit symptoms of Cu toxicity. Copper is mainly accumulated in roots because its root-to-shoot translocation is rather limited (Adrees et al., 2015). As previously observed in *Arabidopsis thaliana*, high Cu concentrations (i.e., 10 and 50 μM Cu) induced a reduction of the primary root growth and an increase of lateral roots development (Lequeux et al., 2010). The reduction of the root length due to Cu toxicity has also been reported in soybean (*Glycine max*; Lin et al., 2007), tomato (*Solanum nigrum* and *Solanum lycopersicum* Al Khateeb and Al-Qwasemeh, 2014), and grapevines (Ambrosini et al., 2015). Moreover, high levels of Cu induced root darkening, a thickening of root tips and a reduction of root hairs (Juang et al., 2012).

The uptake of Cu in plants is mediated by a family of specific transporters, known as Copper Transporter *CTr* or *COPT* gene family. This gene family has already been identified in different plant species and it is formed by a variable number of genes; for example in *A. thaliana* (Sancenón et al., 2003) and *Vitis vinifera* (Martins et al., 2014) five and eight members of the COPT gene family have been identified, respectively. Some pieces of evidence reported that Cu can be also taken up by other bivalent cations transporters, as the Natural Resistance Associated Macrophage Proteins (NRAMP), Zrt- and Irt-like proteins (ZIP), and Iron Related Transporters (IRT) (Wintz et al., 2003; Yruela, 2005; Tsai and Schmidt, 2017). Indeed, IRT has been firstly identified for Fe(II) uptake and, depending on the isoform, for Zn uptake. The IRT-mediated Cu uptake is still controversial. In fact, *A. thaliana* IRT1 is not able to mediate Cu transport (Korshunova et al., 1999), whilst both tomato LeIRT1 and LeIRT2 can transport Cu (Eckhardt et al., 2001). On the other hand, NRAMP have been identified as manganese (Mn), iron (Fe), and Zn transporters (Thomine et al., 2000), whilst NRAMP-mediated Cu uptake has been proved only in mice (Gunshin et al., 1997). Therefore, CTr transporters are the only one specific for Cu transport (Yuan et al., 2011); nonetheless, Cu can also share the uptake pathway with other micronutrients such as Fe, Mn, and Zn, via the activity of the abovementioned bivalent cation transporters. Consistently, when Cu is available at high concentrations, it can compete with the uptake of other micronutrients. Marastoni et al. (2019) reported that high Cu concentrations (i.e., 25 and 50 μM Cu) could affect Mn, Zn, and Fe concentrations in grapevine plant tissues (Marastoni et al., 2019); in addition, the effects provoked by Cu toxicity on micronutrients (i.e., Mn and Zn) concentrations were dependent on the rootstock genotype (i.e., Fercal and 196.17). In particular, the symplastic Mn concentration in root tissue was reduced in Fercal plants, whilst not altered in 196.17 rootstock with increasing Cu concentrations.

Different agronomic practices have been studied to reduce the effects of Cu toxicity improving also the plant nutritional status. Positive effects were reported by Baldi et al. (2018), who proposed the use of phosphate fertilizers to reduce the effects of Cu toxicity on grapevine, while Juang et al. (2014) studied the effects of magnesium (Mg) nutrition in reducing Cu toxicity symptoms. Moreover, also liming was suggested in order to reduce grapevine Cu toxicity symptoms (Ambrosini et al., 2015), obtained by a reduction of Cu concentration in soil solution and an increase in Mg and calcium (Ca) plant uptake, restoring root morphology. The intercropping is also known to improve the resource-use efficiency of crops (Cesco et al., 2006; Brooker et al., 2014; Li et al., 2014) and/or prevent metal toxicity of beneficiary plants (Simões et al., 2012; Brooker et al., 2014; Walker et al., 2014). In particular, our previous study showed how the intercropping of grapevine plants with oat might mitigate Cu toxicity symptoms (Marastoni et al., 2019). The two rootstocks used, Fercal and 196.17, were obtained from the same parental cross (i.e., *Vitis vinifera* × *Vitis berlandieri*), albeit showing a different adaptation to diverse soil conditions; Fercal displays a good tolerance to calcareous soils (i.e., alkaline pH), whilst 196.17 performs better in acidic soils^1^. Interestingly, in these two dramatically different pH conditions of the soils, also the availability of Cu is influenced, implying that the two rootstocks might feature diverse tolerance strategies to excessive Cu concentrations in the growth substrate. In the intercropping experiment, the two rootstocks were differentially affected by the presence of oat, with Fercal performing better than the 196.17. Even the root ionome was modified by the intercropping, and Fercal was reported to better balance root symplast nutrient concentration compared with the 196.17 rootstock (Marastoni et al., 2019).

The different behavior of Fercal and 196.17 rootstocks when exposed to Cu toxicity and/or grown in association with graminaceous plants as oat (i.e., intercropping) suggests that these rootstocks are characterized by different detoxification and competition mechanisms. To elucidate these differences and peculiarities we performed a study combining the alterations of root morphology, Cu concentrations in roots, and shoots with the expression of key genes involved in the uptake of bivalent cations, since both Mn and Fe have shown to have antagonistic and/or synergistic effects with Cu in two grapevine rootstocks (Fercal and 196.17; Marastoni et al., 2019). The ultimate aim of this work was thus to create a possible Cu root uptake model for the two grapevine rootstocks in function of Cu toxicity and the agricultural practices (i.e., intercropping vs. monocropping).

## Materials and Methods

### Plant Material and Growth Conditions

Plant material and growing conditions were conducted as previously described (Marastoni et al., 2019). Briefly, two grapevine rootstocks, Fercal and 196.17 (both *Vitis vinifera* × *Vitis berlandieri*) were provided as small soil-grown cuttings. Plants were removed from soil, roots were carefully cleaned with tap water and subsequently grown in a hydroponic system (1.5 L pots). After 14 days of acclimation in a full nutrient solution [Ca(NO_3_)_2_ 2 mM, KCl 0.1 mM, KH_2_PO_4_ 0.1 mM, K_2_SO_4_ 0.1 mM, MgSO_4_ 0.5 mM, H_3_BO_3_ 10 μM, MnSO_4_ 0.5 μM, CuSO_4_ 0.2 μM, ZnSO_4_ 0.5 μM (NH_4_)_6_Mo_7_O_24_ 0.01 μM, and NaFeEDTA 80 μM] plants were transferred to nutrient solutions supplied with four different Cu concentrations (0.2–5–25–50 μM) for additional 14 days (five biological replicates for each Cu treatment). Copper was provided as CuSO_4_.5H_2_O (Sigma-Aldrich) and the concentration of 0.2 μM was used as control. The composition of the nutrient solution during the treatment was designed following Bravin et al. (2010): briefly, KH_2_PO_4_ concentration was reduced to 0.05 mM and the pH was buffered at 6 with 2-(*N*-morpholino)ethanesulfonic acid (MES)-KOH 1 mM. The nutrient solutions were continuously aerated and replaced every 3–4 days. Plants were grown in a climatic chamber with a light (220 μmol m^−2^ s^−1^)/dark period of 14/10 h at 70% RH.

Both grapevine rootstocks were either grown hydroponically alone (monocropped) or intercropped with oat plants (*Avena sativa* L. cv Fronteira). For the intercropped samples, oat plants were first germinated on a CaSO_4_ 0.5 mM moist paper for 5 days in the dark, then transferred to a hydroponic system with CaSO_4_ 0.5 mM for 24 h to repair possible cell lesions (Hawkesford et al., 2012) and afterward to the full nutrient solution for 6 days. Oat plants were then transferred into pots containing the grapevine rootstocks (15 oat plants per grapevine plant). After 14 days in a full nutrient solution, plants were transferred to nutrient solutions supplied with different Cu concentrations as described above and grown for another 14 days.

### Root Morphology and Cu Composition of Plant Tissues

At harvest, root morphology parameters (total root length, number of tips, root volume, and average root diameter) were determined using the Winrhizo software (EPSON1680, WinRHIZO Pro2003b, Regent Instruments Inc., Quebec, Canada). Afterward, the roots and leaves were separated and dried at 65°C until constant weight was reached, a portion of the root apparatus was frozen in liquid nitrogen and kept at −80°C for gene expression analysis. Roots and leaves tissues were acid digested (HNO_3_ 65% v/v) in a single digestion chamber (SRC) microwave digestion system (UltraWAVE, Milestone, Shelton, CT, United States) and the Cu concentration was determined by Inductively Coupled Plasma Optical Emission Spectroscopy (ICP-OES, Arcos Ametek Spectro, Germany).

### Transporter Gene Sequences Identification

The putative grapevine orthologous genes of *NRAMP* were obtained through a bioinformatics analysis. The amino acidic sequences of *AtNRAMP* (Thomine et al., 2000) and *OsNRAMP* (Takahashi et al., 2011) gene families were used to perform a BlastP analysis using the CRIBI grapevine genome browser^2^. NRAMP are characterized by 12 transmembrane domains (Takanashi et al., 2014), thus the grapevine sequences were screened for the number of transmembrane domains. The prediction of the transmembrane domain was performed with Tmap software (Persson and Argos, 1994). The matching proteins were then screened for phylogenetic relationship between the identified genes and orthologous with DDBJ ClustalW software. The tree visualization was performed with FigTree version 1.4.3. The *VvCTr* genes sequences were obtained by Martins et al. (2012a,b), whereas the *VvIRT1* sequence was obtained by Vannozzi et al. (2017).

### Real-Time RT PCR

The RNA extraction was performed using Spectrum Plant Total RNA Kit (Sigma-Aldrich) after grinding the root tissues in liquid nitrogen. The quality and purity of the samples were obtained spectrophotometrically and by electrophoresis. Then 1 μg of total RNA was subjected to DNAse digestion with 10 U of DNAse RQ1 (Promega) and cDNA was synthetized using ImProm-II Reverse Transcription System (Promega). The amplification primers were designed to be 20 bp long and to obtain an amplicon of 100 bp (Supplementary Table S1). The Elongation Factor 1α and Tubulin housekeeping primers were obtained from Pii et al. (2014). The Real-Time Reverse Transcriptase PCR (Real-Time RT PCR) was performed using the SsoFast EvaGreen Supermix (Bio-Rad) and the Qiagen Rotor Gene Q real-time PCR. The relative expression and the standard error was calculated according to Pfaffl et al. (2002). The amplification efficiency was calculated using LinRegPCR (Ramakers et al., 2003).

### Statistical Analysis

The quantitative analyses have been run on at least three independent biological replicates; for each biological replicate, the quantitative analyses have been run with three technical repetitions. Statistical significance were tested performing Student’s *t*-test and one way ANOVA using GraphPad Prism 5.00.288 for Windows, GraphPad Software, San Diego, CA, United States.

## Results

### Root Morphology and Architecture

**FIGURE 1:**
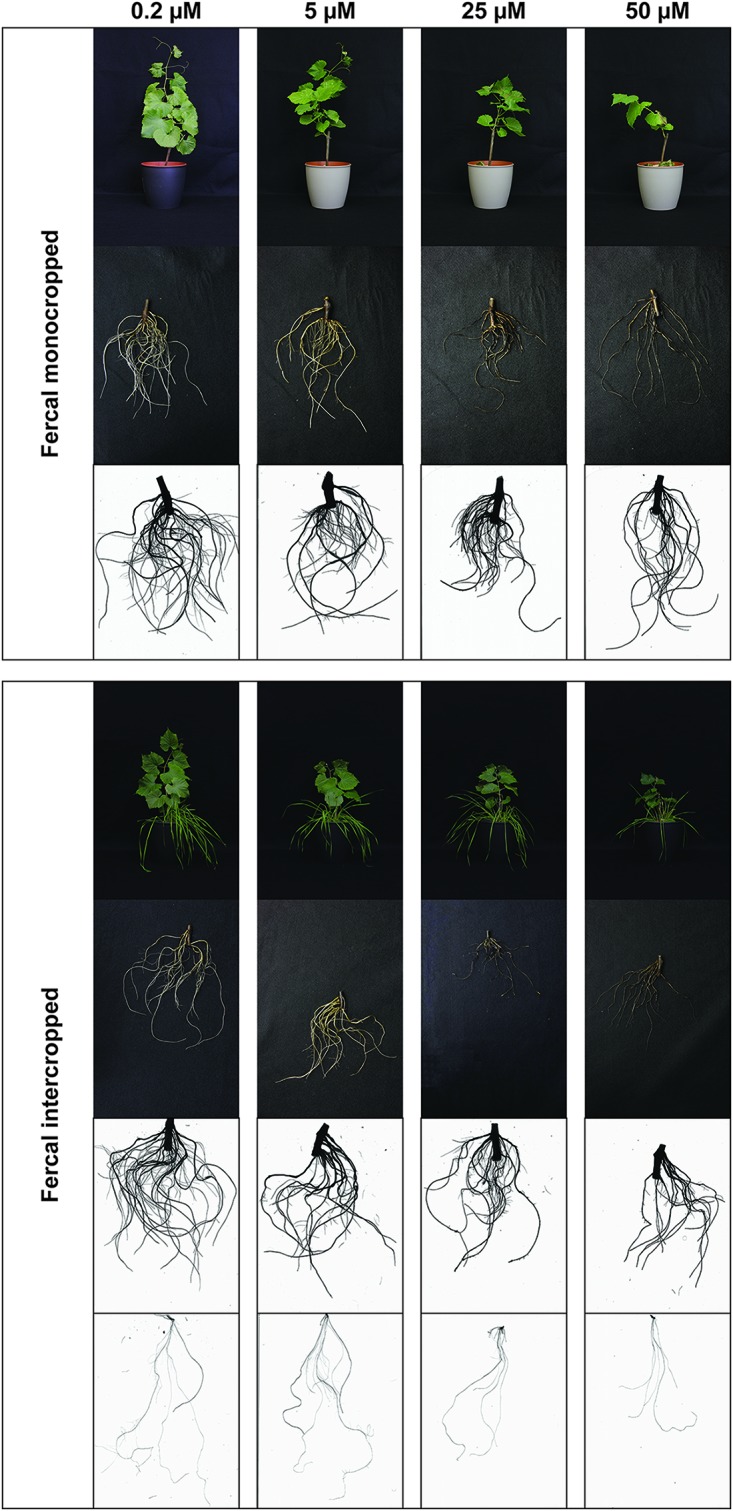
Phenotype of Fercal rootstock plants grown at different Cu concentrations. Representative pictures of Fercal rootstocks (shoots, roots, and a representative WinRhizo Scan of the root apparatus) in both growing conditions (mono- and intercropping) at different concentrations of Cu (0.2–5–25–50 μM). When presenting intercropping condition, also a representative WinRhizo Scan of oat root apparatus is shown. Pictures have been taken at harvest, i.e., 14 days after Cu treatment.

**FIGURE 2:**
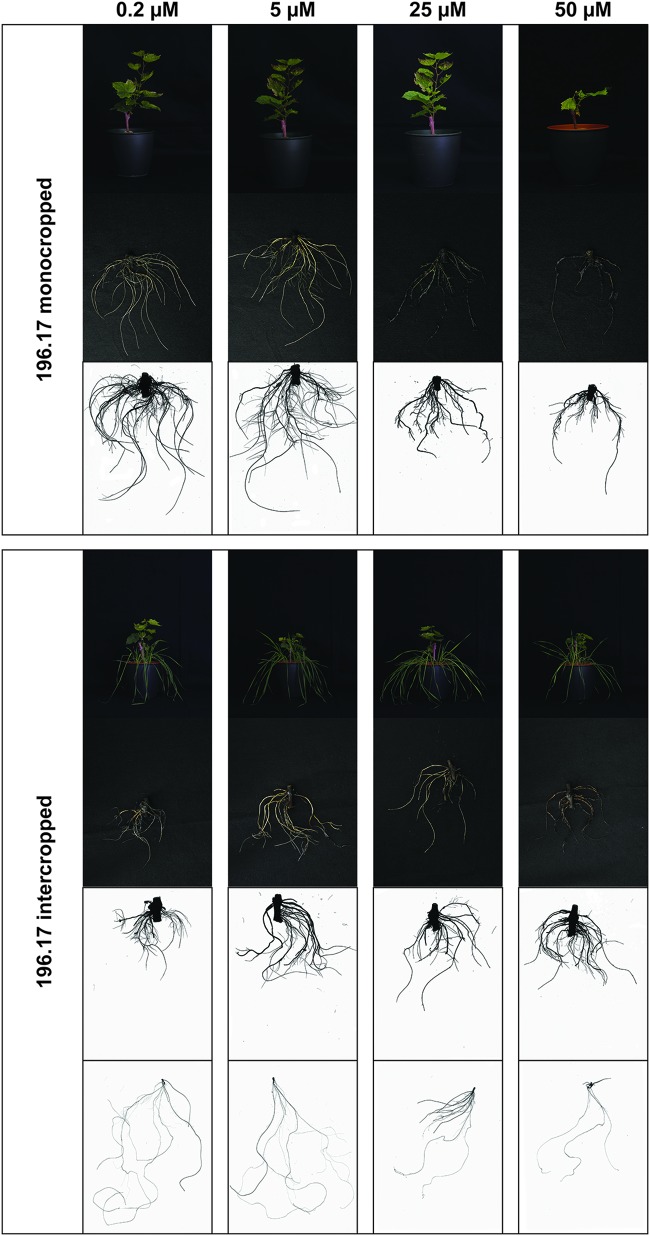
Phenotype of 196.17 rootstock plants grown at different Cu concentrations. Representative pictures of 196.17 rootstocks (shoots, roots, and a representative WinRhizo Scan of the root apparatus) in both growing conditions (mono- and intercropping) at different concentrations of Cu (0.2–5–25–50 μM). When presenting intercropping condition, also a representative WinRhizo Scan of oat root apparatus is shown. Pictures have been taken at harvest, i.e., 14 days after Cu treatment.

**FIGURE 3:**
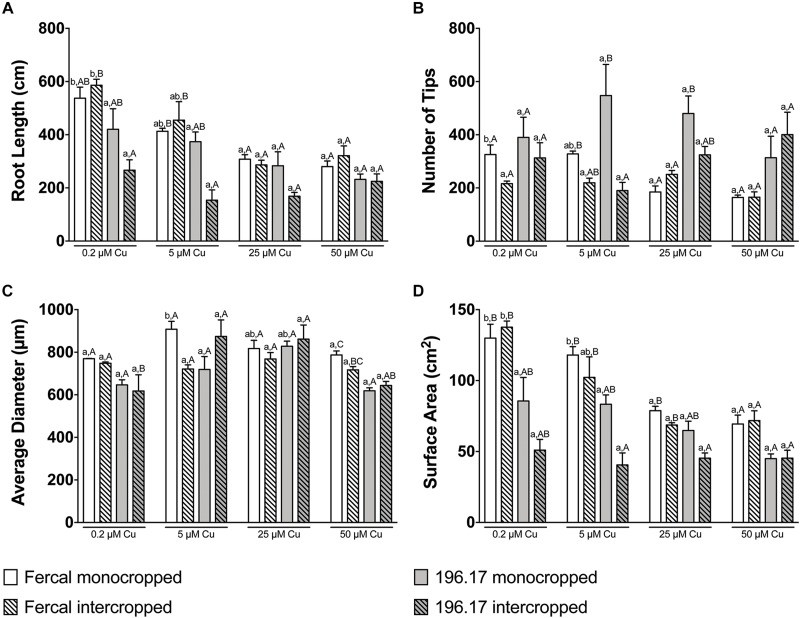
Root architecture parameters of Fercal and 196.17 rootstock plants, either monocropped or intercropped with oat, ad exposed to increasing concentration of Cu. **(A)** Total root length. The statistical significance of the whole dataset was tested by a three-way ANOVA test: Cu concentration *p* < 0.0001; Genotype *p* < 0.0001; Cropping system *p* = 0.0155; Cu concentration × Genotype *p* = 0.0189; Cu concentration × Cropping system *p* = 0.2533; Genotype × Cropping system *p* = 0.0002; Cu concentration × Genotype × Cropping system *p* = 0.2004. **(B)** Total number of tips. The statistical significance of the whole dataset was tested by a three-way ANOVA test: Cu concentration *p* = 0.3870; Genotype *p* < 0.0001; Cropping system *p* = 0.0033; Cu concentration × Genotype *p* = 0.3179; Cu concentration × Cropping system *p* = 0.0051; Genotype × Cropping system *p* = 0.1043; Cu concentration × Genotype × Cropping system *p* = 0.0617. **(C)** Average diameter. The statistical significance of the whole dataset was tested by a three-way ANOVA test: Cu concentration *p* < 0.0001; Genotype *p* = 0.0107; Cropping system *p* = 0.3830; Cu concentration × Genotype *p* = 0.0063; Cu concentration × Cropping system *p* = 0.9896; Genotype × Cropping system *p* = 0.0025; Cu concentration × Genotype × Cropping system *p* = 0.0229. **(D)** Total surface area. The statistical significance of the whole dataset was tested by a three-way ANOVA test: Cu concentration *p* < 0.0001; Genotype *p* < 0.0001; Cropping system *p* = 0.0008; Cu concentration × Genotype *p* = 0.0003; Cu concentration × Cropping system *p* = 0.0681; Genotype × Cropping system *p* = 0.0132; Cu concentration × Genotype × Cropping system *p* = 0.2807. Data have been collected at harvest (14 days after Cu treatment) and are reported as means (±SE), *n* = 5. Different letters indicate significantly different values as determined using one-way ANOVA with Tukey *post hoc* tests (*p* < 0.05). Lower-case letters account for statistical analyses of the same cropping system exposed to different Cu concentrations. Upper-case letters account for statistical analyses of the different cropping system exposed to the Cu concentration.

**FIGURE 4:**
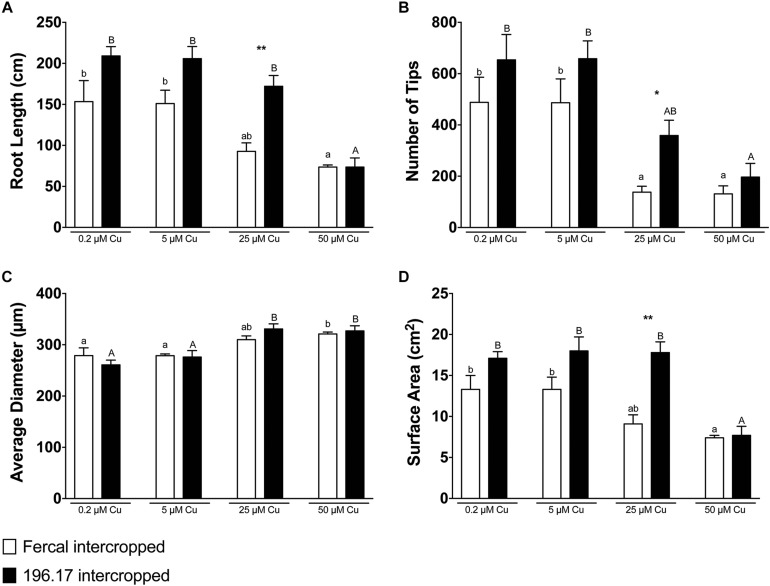
Root architecture parameters of oat, intercropped with either Fercal or 196.17 rootstock plants, ad exposed to increasing concentration of Cu. **(A)** Total root length. **(B)** Total number of tips. **(C)** Average diameter. **(D)** Total surface area. Data have been collected at harvest (14 days after Cu treatment) and are reported as means (±SE), *n* = 5. Different letters indicate significantly different values as determined using one-way ANOVA with Tukey *post hoc* tests (*p* < 0.05). Lower-case letters account for statistical analyses of oat plants intercropped with Fercal rootstock plants and exposed to different Cu concentrations. Upper-case letters account for statistical analyses of oat plants intercropped with Fercal rootstock plants and exposed to different Cu concentrations. Within the same Cu concentration condition, the difference between Fercal-intercropped oat and 196.17-intercropped oat was tested through a Students *t*-test (^*^p < 0.05; ^∗∗^*p* < 0.01).

The high Cu concentrations affected the root morphology of both grapevine rootstocks, particularly Fercal, and oat plants (Figures 1–4), albeit to different extent. In particular, a darkening of the roots was observed with increasing Cu concentrations in both grapevine rootstocks independently from the growing condition (Figures 1, 2). Furthermore, Cu caused a thickening and a modification of the structure (bumping surface) of root tips (Supplementary Figure S1). This Cu toxicity symptom was visible only in 25 μM Cu-treated Fercal plants grown in both monocropping and intercropping systems (Figure 1). Differently, monocropped 196.17 rootstock showed a root tips modification when exposed to 5 and 25 μM of Cu, whilst the intercropped rootstocks displayed the same symptoms at the highest Cu concentrations (i.e., 25 and 50 μM) (Figure 2). Furthermore, the concentration of 50 μM Cu in the nutrient solutions led to necrotic roots in all the growing conditions in both rootstocks (Figures 1, 2).

The root architecture of monocropped Fercal rootstocks treated with 25 and 50 μM Cu revealed a decreased root length and number of root tips compared to the control (Figures 3A,B). Although intercropped Fercal rootstocks grown at 25 and 50 μM Cu showed a decreased root length compared to the control, the number of tips was not modified by the increasing Cu concentrations (Figures 3A,B) In addition, the surface area decreased in mono- and intercropped Fercal plants cultivated at 25 and 50 μM Cu. However, when comparing mono- and intercropped Fercal plants, within each Cu concentrations used in the experiment, any statistically significant difference in root length, number of tips and root surface area could be highlighted (Figure 3). Moreover, no differences of root morphology parameters of both mono- and intercropped 196.17 rootstocks were observed with increasing Cu concentrations (Figure 3).

Comparing the two rootstocks, in control conditions (0.2 μM Cu) intercropped 196.17 rootstocks showed a significant decrease in the root length and in the root surface area as compared to intercropped Fercal (Figures 3A,D). At 50 μM Cu, monocropped Fercal reported a higher root diameter compared to the monocropped 196.17, whilst no differences were detected in the root length, in the number of tips and in the surface area (Figure 3). On the other hand, at 25 μM Cu Fercal rootstocks, regardless mono- and intercropping growing system, displayed a lower number of tips and a higher surface area as compared to mono- and intercropped 196.17 rootstock (Figures 3B,D).

Oat plants reduced their root length, number of tips and root surface at high Cu concentrations (i.e., 25 and 50 μM Cu) independently from the rootstock they are intercropped with (Figure 4). However, at 25 μM Cu both the number of tips and the surface area of Fercal-intercropped oat are lower than those detected in 196.17-intercropped oat plants (Figures 4B,D). This difference was not present at 50 μM Cu, however, oat plants grown at this Cu concentration showed an increased root diameter independently from the intercropped rootstock (Figure 4C).

### Cu Concentration in Plant Tissues

**FIGURE 5:**
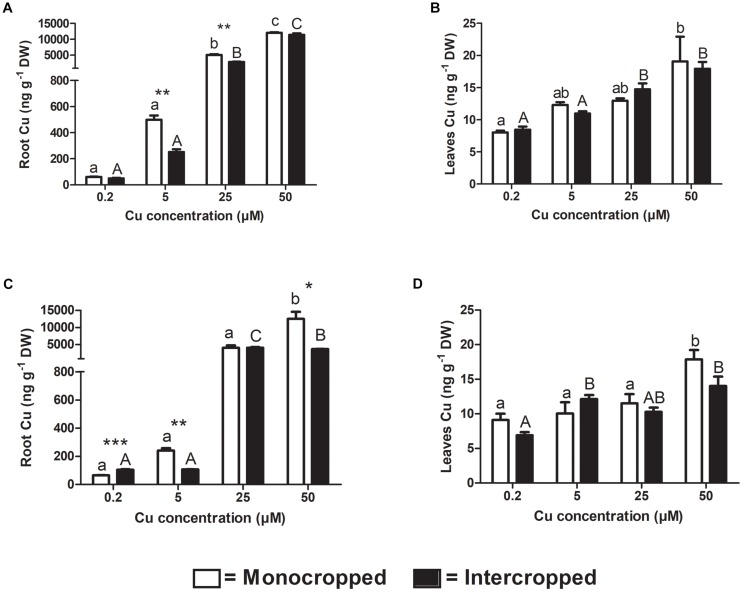
Analyses of Cu concentration in grapevine plant tissues. Copper concentration detected in roots **(A)** and leaves **(B)** of both mono- and intercropped Fercal rootstock plants, and in roots **(C)** and leaves **(D)** of both mono- and intercropped 196.17 rootstock plants. The data are reported as means ± SE of three biological replicates. Different letters indicate significantly different values as determined using one-way ANOVA with Tukey *post hoc* tests (*p* < 0.05). Lower case letters indicate the statistical significance for monocropped plants in the different Cu concentrations, whilst upper case letters indicate the statistical significance for intercropped plants grown in the different Cu concentrations. Stars indicate the significance (Student’s *t*-test) between the Cu concentrations detected in mono- and intercropped rootstock plants (^*^p < 0.05; ^∗∗^*p* < 0.01; ^∗∗∗^*p* < 0.001).

As expected, Cu accumulation in plant tissues followed the increasing Cu concentration in the nutrient solution in all growing conditions of both Fercal and 196.17 rootstocks (Figure 5). The roots were the main targets for Cu accumulation. In fact, Cu translocation to the shoots was very limited, being the Cu concentration up to 700-fold lower than in the grapevine roots treated with 25 and 50 μM Cu (Figure 5). Furthermore, the Cu increase was more pronounced for the root tissues (Fercal: +1109, 8193, 5871%, and 196.17: +276, 6100, and 19508% vs. the control at 5, 25, and 50 μM, respectively) compared to the shoot (Fercal: +53, 61, 138%, and 196.17: +10, 26, 96%, vs. the control at 5, 25, and 50 μM, respectively).

Comparing the two growing conditions, i.e., monocropping and intercropping, a reduction in Cu accumulation might be inferred, at least in some cases (Figure 5). In detail, for the Fercal plants, the reduction resulted significant only in the root tissues and only for the intermediate Cu concentrations applied (5 and 25 μM). For the intercropped 196.17 rootstock, a reduction of root Cu concentration was observed only at 5 and 50 μM Cu compared to the control, whereas at 0.2 μM Cu intercropped plants displayed a higher Cu concentration compared to the monocropped 196.17 (Figure 5).

### *CTr* Genes Expression

**FIGURE 6:**
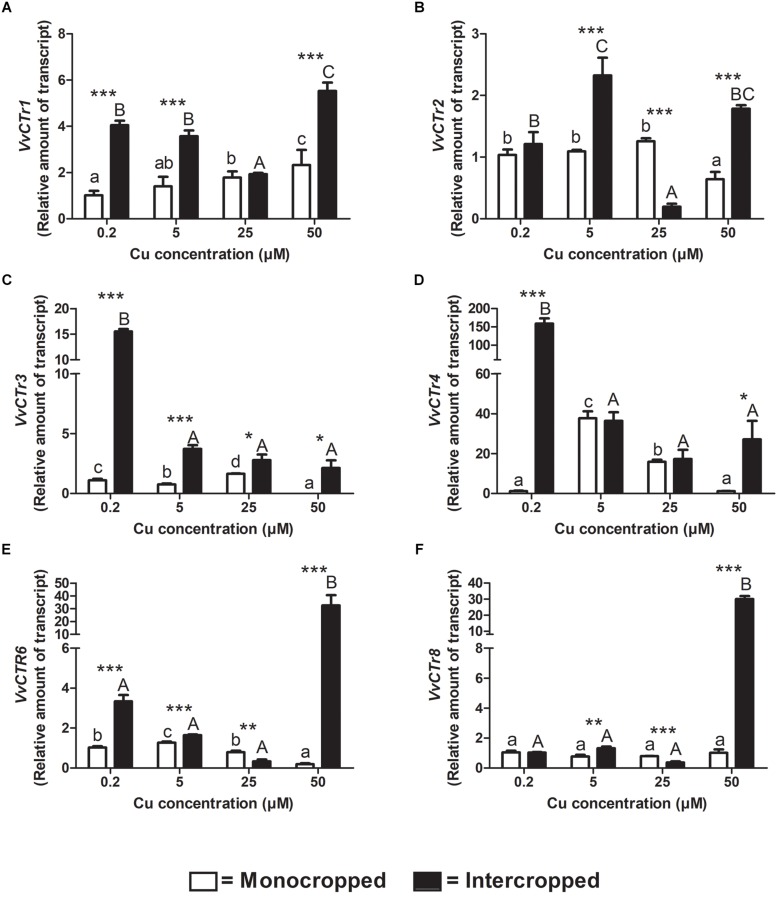
Quantitative real time RT-PCR analyses of *VvCTr* genes in Fercal rootstock plants. Root apex genes expression of *VvCTr1* **(A)**, *VvCTr2* **(B)**, *VvCTr3* **(C)**, *VvCTr4* **(D)**, *VvCTr6* **(E)**, and *VvCTr8* **(F)** determined in mono- and intercropped Fercal rootstocks. Transcripts levels are reported as relative amount of transcripts referred to the gene expression of monocropped rootstocks grown at 0.2 μM Cu. The data are reported as means ± SE of three biological replicates. Different letters indicate significantly different values as determined using one-way ANOVA with Tukey *post hoc* tests (*p* < 0.05). Lower case letters indicate the statistical significance for monocropped plants in the different Cu concentrations, whilst upper case letters indicate the statistical significance for intercropped plants grown in the different Cu concentrations. Stars indicate the significance (Student’s *t*-test) between the gene expression of mono- and intercropped rootstocks (^*^ *p* < 0.05; ^∗∗^ *p* < 0.01; ^∗∗∗^ *p* < 0.001).

**FIGURE 7:**
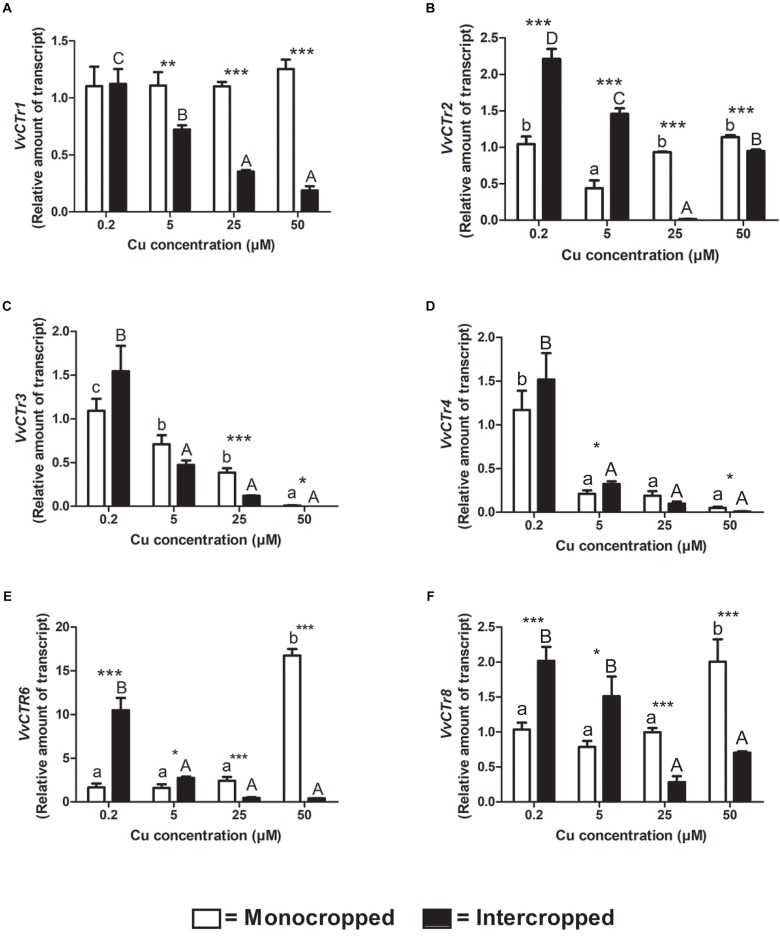
Quantitative real time RT-PCR analyses of *VvCTr* genes in 196.17 rootstock plants. Root apex genes expression of *VvCTr1* **(A)**, *VvCTr2* **(B)**, *VvCTr3* **(C)**, *VvCTr4* **(D)**, *VvCTr6* **(E)**, and *VvCTr8* **(F)** determined in mono- and intercropped 196.17 rootstocks. Transcripts levels are reported as relative amount of transcripts referred to the gene expression of monocropped rootstock grown at 0.2 μM Cu. The data are reported as means ± SE of three biological replicates. Different letters indicate significantly different values as determined using one-way ANOVA with Tukey *post hoc* tests (*p* < 0.05). Lower case letters indicate the statistical significance for monocropped plants in the different Cu concentrations, whilst upper case letters indicate the statistical significance for intercropped plants grown in the different Cu concentrations. Stars indicate the significance (Student’s *t*-test) between the gene expression of mono- and intercropped rootstocks (^*^p < 0.05; ^∗∗^*p* < 0.01; ^∗∗∗^*p* < 0.001).

The components of the *VvCTr* gene family were already identified by Martins et al. (2012a, b) in *V. vinifera* genome. The expression analyses of the *CTr* genes revealed that, at 0.2 μM of Cu, the most expressed genes are the *VvCTr1*, *VvCTr2*, and *VvCTr3* in roots of the Fercal rootstock, and the *VvCTr1* and *VvCTr3* in the roots of the 196.17 rootstock (Supplementary Figure S3). The expression of *VvCTr1* increased in roots of monocropped Fercal rootstocks with increasing Cu concentrations, whereas it was constitutively expressed in the roots of the 196.17 rootstocks (Figures 6A, 7A). The expression of *VvCTr1* was up-regulated at all the Cu concentration, except 25 μM Cu, in the roots of the intercropped Fercal rootstock in comparison to monocropped plants (Figure 6A); on the contrary, the expression of *VvCTr1* was not affected by the intercropping in 196.17 rootstock at 0.2 μM Cu, whereas it decreased with the increasing Cu concentrations (Figure 7A). The *VvCTr2* expression was unchanged in the roots of monocropped Fercal rootstock, albeit a significant reduction was observed in plants grown at 50 μM Cu (Figure 6B). The intercropping induced a *VvCTr2* up-regulation at 5 and 50 μM Cu in Fercal plants and a gene down-regulation at 25 μM Cu (Figure 6B). The roots of the 196.17 rootstock did not show a high expression of the *VvCTr2* gene at 0.2 μM Cu (Supplementary Figure S3): it remained almost constant in monocropped plants, whilst it was progressively repressed with increasing Cu concentration in intercropped plants (Figure 7B).

In monocropped Fercal rootstocks, *VvCTr3* expression was reduced as compared to *VvCTr1*, whereas an opposite trend was detected in monocropped 196.17 rootstock (Supplementary Figure S3). The increase in Cu concentrations induced the up-regulation of *VvCTr3* in monocropped Fercal rootstocks, except at 50 μM Cu (Figure 6C), whilst it reduced the expression of *VvCTr3* in the roots of monocropped 196.17 rootstocks (Figure 7C). The intercropping strongly induced the expression of *VvCTr3* in Fercal plants (Figure 6C); in 196.17 rootstock, the intercropping did not modify *VvCTr3* transcription at 0.2 and 5 μM Cu whilst it caused a significant reduction in the gene expression at 25 and 50 μM Cu compared to the same monocropped rootstock (Figure 7C). The *VvCTr4*, *VvCTr6*, and *VvCTr8* genes were less expressed both in monocropped Fercal and 196.17 rootstocks at 0.2 μM Cu (Supplementary Figure S3). Their expression in monocropped Fercal plants remained low at all the Cu concentrations (Figures 6D–F); the intercropping induced *VvCTr4* at 0.2 μM of Cu and *VvCTr6* and *VvCTr8* at 50 μM Cu (Figures 6D–F). The mRNA levels of *VvCTr4*, *VvCTr6*, and *VvCTr8* genes in 196.17 rootstock showed a conserved pattern: the intercropping induced a significant up-regulation of genes at the lower Cu concentrations (i.e., 0.2 and 5 μM), and a strong down-regulation at the higher ones (i.e., 25 and 50 μM) (Figures 7D–F).

### NRAMP Genes Expression

The *NRAMP* gene family has never been characterized before in *V. vinifera* genome; thus, to reach this objective, a bioinformatic approach was undertaken and six putative sequences similar to AtNRAMP and OsNRAMP have been identified. Among these, three transcripts were found to feature the characteristic 12 transmembrane domains and cluster with the members of the *AtNRAMP* and *OsNRAMP* gene family. These three transcripts were named *VvNRAMP1* (VIT_209s0070g00210), *VvNRAMP2* (VIT_218s0001g09490), and *VvNRAMP3* (VIT_207s0129g00620) because they clustered with *AtNRAMP1*, *AtNRAMP2*, and *AtNRAMP3*, respectively (Supplementary Figure S2).

**FIGURE 8:**
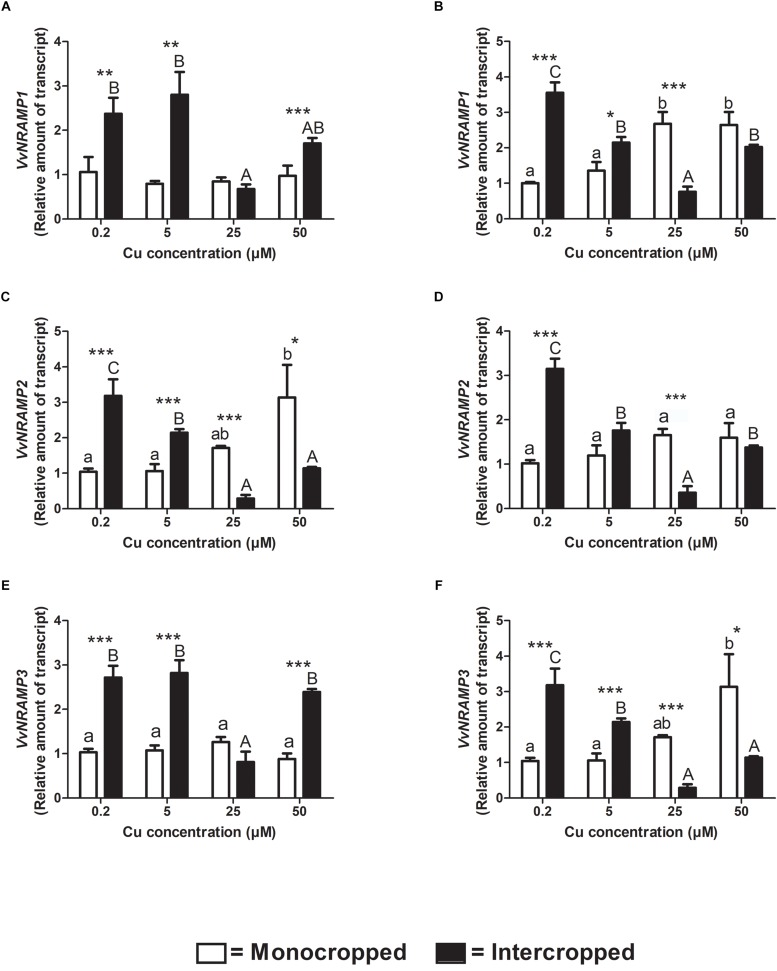
Quantitative real time RT-PCR analyses of *VvNRAMP* genes in rootstock plants. Root apex genes expression of *VvNRAMP1*, *VvNRMP2*, and *VvNRMP3* determined in mono- and intercropped Fercal **(A,C,E)** and 196.17 **(B,D,F)** rootstocks. Transcripts levels are reported as relative amount of transcripts referred to the gene expression of monocropped rootstock grown at 0.2 μM Cu. The data are reported as means ± SE of three biological replicates. Different letters indicate significantly different values as determined using one-way ANOVA with Tukey *post hoc* tests (*p* < 0.05). Lower case letters indicate the statistical significance for monocropped plants in the different Cu concentrations, whilst upper case letters indicate the statistical significance for intercropped plants grown in the different Cu concentrations. Stars indicate the significance (Student’s *t*-test) between the gene expression of mono- and intercropped rootstocks (^*^p < 0.05; ^∗∗^*p* < 0.01; ^∗∗∗^*p* < 0.001).

*VvNRAMP1* is differentially modulated in the two different rootstocks (Figures 8A,B). Fercal plants did not modulate the transcription depending on Cu concentration in the hydroponic solution (Figure 8A). On the contrary, the expression of *VvNRAMP1* increased in roots of the 196.17 rootstock at 25 and 50 μM of Cu (Figure 8B). When intercropped, both the rootstocks up-regulated *VvNRAMP1*expression at 0.2 and 5 μM Cu and down-regulated it at 25 μM Cu (Figures 8A,B). At 50 μM Cu, the transcription of *VvNRAMP1* was increased in roots of the intercropped Fercal rootstock and reduced in those of the intercropped 196.17 rootstock. *VvNRAMP2* was only slightly modulated in roots of monocropped Fercal rootstocks with increasing Cu concentrations (Figure 8C). The expression of *VvNRAMP2* was enhanced in roots of intercropped Fercal rootstocks at 0.2 and 5 μM Cu, while it was reduced at 25 and 50 μM Cu (Figure 8C). The roots of intercropped 196.17 rootstocks showed an over-expression of *VvNRAMP2* at 0.2 μM Cu, no changes at 5 and 50 μM Cu and a down-regulation at 25 μM Cu (Figure 8D).

*VvNRAMP3* reported the same expression pattern as *VvNRAMP1* for both the rootstocks and cultivation systems (Figures 8A,B,E,F).

### IRT Genes Expression

**FIGURE 9:**
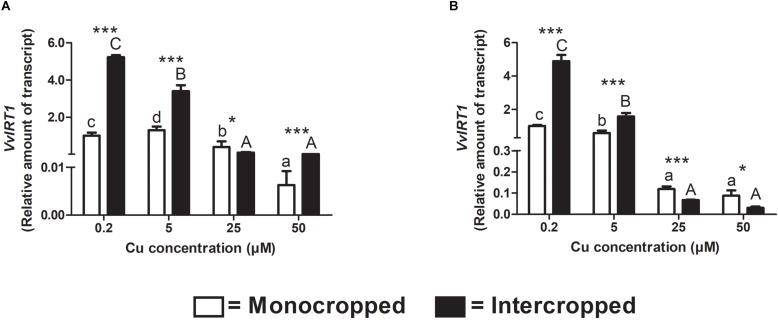
Quantitative real time RT-PCR analyses of *VvIRT1* genes in rootstock plants. Root apex gene expression of *VvIRT1* determined in mono- and intercropped Fercal **(A)** and 196.17 **(B)** rootstocks. Transcripts levels are reported as relative amount of transcripts referred to the gene expression of monocropped rootstock grown at 0.2 μM Cu. The data are reported as means ± SE of three biological replicates. Different letters indicate significantly different values as determined using one-way ANOVA with Tukey *post hoc* tests (*p* < 0.05). Lower case letters indicate the statistical significance for monocropped plants in the different Cu concentrations, whilst upper case letters indicate the statistical significance for intercropped plants grown in the different Cu concentrations. Stars indicate the significance (Student’s *t*-test) between the gene expression of mono- and intercropped rootstocks (^*^p < 0.05; ^∗∗^*p* < 0.01; ^∗∗∗^*p* < 0.001).

The *VvIRT1* has already been identified in *V. vinifera* genome and it was reported to be implicated in the response toward Fe-deficiency conditions (Vannozzi et al., 2017). In our experimental conditions, the expression of *VvIRT1* resulted down-regulated in roots of monocropped Fercal and 196.17 rootstocks with increasing Cu concentrations (Figures 9A,B). Intercropping led to an increased *VvIRT1* expression in both rootstocks at 0.2 and 5 μM Cu and to a decreased expression at 25 μM Cu. Fercal intercropped induces the expression of *VvIRT1* at 50 μM Cu compared to the monocropped rootstock; on the contrary, the 196.17 intercropped rootstock showed a repression of *VvIRT1* expression at 50 μM Cu (Figures 9A,B).

## Discussion

Different strategies and farming systems have been studied to reduce the effect of Cu toxicity on plants (Mackie et al., 2012). A previous study demonstrated that intercropping grapevine plants with an herbaceous species, as oat, alleviates Cu toxicity (Marastoni et al., 2019), yet the positive effects are rootstock-type dependent. Since Cu toxicity affected also the nutrient uptake, in this work we analyzed both root morphology and the transcription levels of divalent cation transporters, since both Mn and Fe have shown to have antagonistic and/or synergistic effects with Cu in two grapevine rootstocks (Fercal and 196.17; Marastoni et al., 2019). For this study, Fercal and 196.17 rootstocks were chosen since they are obtained from the same parental cross (i.e., *Vitis vinifera* × *Vitis berlandieri*), yet being selected for different adaptation strategies to diverse soil conditions. In particular, Fercal displays a high tolerance to active lime in the soil (i.e., alkaline soils), whereas 196.17 performs better in acidic soils, featuring very low active lime (see footnote 1). Interestingly, in these two dramatically different pH conditions of the soils, also the availability of Cu is influenced, implying that the two rootstocks might feature diverse tolerance strategies to excessive Cu concentrations in the growth substrate.

### Root Architecture of Mono- and Intercropped Rootstocks

At root level, grapevine plants showed a phenotype that might be ascribable to Cu toxicity symptoms. In fact, both mono- and intercropped Fercal and 196.17 rootstocks reduced theirs shoot and root growth, and roots became darker with increasing Cu concentration (Figures 1, 2). Moreover, the bumping of the root tips was observed in both rootstocks and growing conditions at high Cu concentration (25 and 50 μM Cu; Supplementary Figure S1). The thickening of the root apex was already reported in grapevine plants grown at high Cu concentrations (Ambrosini et al., 2015). The same authors also observed a reduced root growth as result of an inhibition of cell division at the root apex (Ambrosini et al., 2015). Accordingly, both mono- and intercropped Fercal plants reduced their root length at 25 and 50 μM Cu (Figure 3A), whereas the increasing Cu concentrations did not significantly alter the root length of the 196.17 rootstock in both growing conditions (Figure 3A), thus suggesting a rootstock-dependent sensitivity to root length modifications (Figure 3). The same has been observed for the number of tips and the surface area. Comparing the two intercropped rootstocks, they had a different root length and surface area. Indeed, intercropped Fercal rootstocks had a higher root length and surface area compared to the intercropped 196.17 rootstocks. A larger root apparatus could be considered a mechanism to overcome metal toxicity and the induced nutrient deficiency, since it allows plants to accumulate higher concentrations of metal ions in root apoplast (Horst et al., 2010) and to explore a bigger soil volume, respectively. As already hypothesized in Marastoni et al. (2019), the data hereby presented demonstrate that Fercal is well adapted to grow in a competing environment (i.e., direct competition with other plant species for mineral nutrients), whereas 196.17 do not properly respond to the induced nutrient deficiencies. Fercal accumulates more Cu in its root tissues compared to the 196.17 (Figures 5A,C) and the intercropping reduces the root Cu concentration (Figures 5A,C).

### Gene Expression in Monocropped Grapevine Rootstocks

**FIGURE 10:**
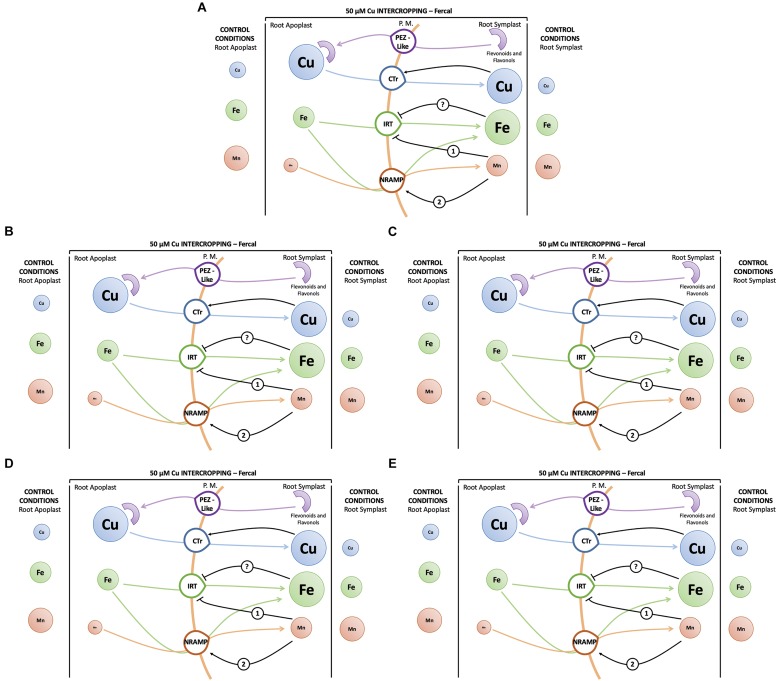
Hypothesis of a root uptake model of cations: **(A)** Model for monocropped Fercal and 196.17 rootstocks at high Cu concentrations (i.e., 25 and 50 μM Cu). **(B)** Model for intercropped Fercal rootstocks in low Cu toxicity (5 μM Cu). **(C)** Model for intercropped Fercal rootstocks at high Cu toxicity (50 μM Cu). **(D)** Model for intercropped 196.17 rootstocks in low Cu toxicity (5 μM Cu). **(E)** Model for intercropped 196.17 rootstock at high Cu toxicity (50 μM Cu). Size of circles represent the estimated ion concentration in the single compartments. The concentrations data used for the development of the model are taken from this work and from Marastoni et al. (2019). Numbers indicate the sequence of events, the rootstock name on the arrows specify that the description is characteristic for the specified rootstock and not for the other.

To better clarify this phenomenon, it was necessary to study how the different growing conditions could affect the expression of *VvCtr*, *VvNRAMP*, and *VvIRT1* transporter genes. Both rootstocks expressed the genes *VvCTr2* and *VvCTr3* (Figure 6) that cluster with *AtCOPT1* (Martins et al., 2012a), which has been shown to be mainly expressed at the root apex in *A. thaliana* and likely playing a role in Cu uptake (Puig, 2014). Considering the expression pattern of *VvCTr2* and *VvCTr3*, it could be hypothesized that *VvCTr2* and *VvCTr3* might be involved in the Cu uptake in Fercal and 196.17 rootstocks, respectively. Furthermore, roots of monocropped Fercal rootstocks do not modulate *VvCTr2* with increasing Cu concentration (i.e., 5 and 25 μM Cu) (Figure 6B), whereas *VvCTr3* is down-regulated in roots of monocropped 196.17 rootstocks with increasing Cu concentrations (Figure 7C). However, regardless *VvCTr* genes expressions, the Cu concentration increased in both monocropped rootstocks root tissues (Figures 5A,C). This might suggest that Cu, beside *VvCTr* transporters, can use other mediators to reach the root symplast; indeed, *VvNRAMP* and *VvIRT1* could thereby be valuable candidates to transport Cu. Three members of the *VvNRAMP* gene family have been identified in this work; among them, *VvNRAMP1* clustered with *AtNRAMP1* (Supplementary Figure S2), which has been reported to take up both Mn and Fe and has been identified as the only transporter of Mn in *A. thaliana* (Cailliatte et al., 2010). *VvNRAMP2* clustered with *AtNRAMP2* (Supplementary Figure S2), which is localized in the Trans-Golgi (Gao et al., 2018), whereas *VvNRAMP3* clustered with *AtNRAMP3* and *AtNRAMP4* (Supplementary Figure S2), which are located at the tonoplast (Lanquar et al., 2005). Consequently, the phylogenetic analysis suggested that *VvNRAMP1* could be the transporter involved in Mn uptake and potentially implicated in Cu(II) uptake. Whilst *VvNRAMP1* is not modulated in roots of monocropped Fercal rootstocks with increasing Cu concentrations (Figure 8A), roots of monocropped 196.17 rootstocks increased *VvNRAMP1* expression at 25 and 50 μM Cu (Figure 8B). This up-regulation is most likely due to a Cu-induced Mn deficiency in the root apoplast (Marastoni et al., 2019). Since a synergistic relationship of Fe with Cu has been reported (Marastoni et al., 2019), we also studied the transcriptional modulation of *VvIRT1* in our experimental model. *VvIRT1* expression decreased in both monocropped rootstocks at high Cu concentrations (25 and 50 μM Cu; Figures 9A,B). The reduction of *VvIRT1* expression could be induced to prevent Fe toxicity due to the high root Fe concentrations reported for both rootstocks (Marastoni et al., 2019). A study carried out on *A. thaliana* plants revealed that Mn deficiency could induce a down regulation of *AtIRT1* with an increase of root Fe concentration (Yang et al., 2008). Cailliatte et al. (2010) also reported that Mn deficiency could induce *AtNRAMP1*, albeit being its modulation slow. Moreover, they also demonstrated that Fe can be transported also by *AtNRAMP1* (Cailliatte et al., 2010). A similar gene modulation pattern was observed here in the roots of 196.17 rootstocks and this is in good agreement with previous ionomic study that reported an increase in Fe concentration in the root tissues of plants grown in the same conditions (Marastoni et al., 2019). Consequently, we could hypothesize that the 196.17 rootstocks reduce the expression of *VvCTr3* when exposed to high Cu concentrations. Based on these observation, we develop an hypothetical uptake model for bivalent cation (Cu, Fe, and Mn) in monocropped grapevines (Figure 10A): the low Mn concentration in the root apoplast (Marastoni et al., 2019) could be perceived as a reduced Mn availability that can firstly down-regulate *VvIRT1* expression in monocropped 196.17 as reported in *A. thaliana* (Yang et al., 2008). This could be a possible consequence of the high Fe concentration in root symplast (Marastoni et al., 2019). Secondly, the Cu-induced Mn deficiency could induce *VvNRAMP1* at high Cu concentrations. Differently, Fercal rootstocks do not modulate *VvCTr2* and *VvNRAMP1* with increasing Cu concentration but down regulate *VvIRT1* (Figures 6B, 8A, 9A). These different expression patterns could corroborate the better performance of the 196.17 rootstock at high Cu concentrations compared to the Fercal rootstock. Indeed the two rootstocks have the same parental grapevines (*Vitis vinifera* × *Vitis berlanderi*) and have been selected to grow in acidic (196.17) and calcareous (Fercal) soils (Pavloušek, 2013). The available fraction of nutrients, especially the cationic micronutrients, is dependent on soil pH. Therefore, specifically in monocropping systems, 196.17 could be expected to better manage high level of Cu, in terms of a lower accumulation of metal at root level, and the induced nutrient unbalances (Mn deficiency). On the contrary, Fercal is adapted to grow in soil with low nutrient availability, thus preferring the upregulation of micronutrients uptake rather than the response to Cu toxicity. Indeed, Fercal accumulates high concentration of Cu in the root tissues.

### Gene Expression in Intercropped Grapevine Rootstocks

Intercropped grapevines showed a different behavior depending on the Cu toxicity (i.e., low – 5 μM, medium – 25 μM, and high – 50 μM). At 5 μM Cu, intercropped Fercal showed the over-expression of *VvCTr2*, *VvNRAMP1*, and *VvIRT1* (Figures 6B, 8A, 9A) that might be ascribable to a possible nutrient competition with oat (Figure 10B). At 50 μM Cu, intercropped Fercal induced *VvCTr2* expression compared to the monocropped Fercal. Moreover, *VvNRAMP1* and *VvIRT1* are induced at high Cu concentrations (Figures 8A, 9A). However, it has been shown that at 50 μM Cu the apoplastic Mn concentration in intercropped Fercal is not detectable and it is lower than in the monocropping growing condition (Marastoni et al., 2019); such drop in the Mn concentration might induce a Mn-deficincy-like response thus causing the enhancement of *VvNRAMP1* expression. These observations might suggest that Fercal rootstock cannot cope with Cu toxicity and Cu-induced Mn deficiency at the same time, thus preferring the up-regulation of cationic transporters to take up Mn rather than the down-regulation of *VvCTr2* to avoid Cu toxicity. However, it has been demonstrated that Fercal also up-regulates the *VvPEZ-like* genes releasing higher concentration of phenolic compounds when intercropped with oat, indicating that Fercal might adopt an external detoxification strategy (Marastoni et al., 2019; Figure 10C).

At low Cu concentrations (0.2 and 5 μM), intercropped 196.17 rootstocks have shown to accumulate more cations in the root apoplast than the monocropped ones (Marastoni et al., 2019). This could suggest that when intercropped, the 196.17 rootstocks modify the composition of the root apoplast (e.g., the pectin methylation degree) as reported for several plants subjected to metal toxicity (Mimmo et al., 2009). At the same time, at low Cu concentrations, roots of the intercropped 196.17 rootstock induces the expression of *VvCTr2* (mostly at 5 μM Cu), *VvNRAMP1* and *VvIRT1* (Figure 10D).

At high Cu concentrations (50 μM), roots of intercropped 196.17 rootstocks down-regulated *VvCTr3* (*p* < 0.0001), up-regulated *VvNRAMP1* (*p* < 0.0001) and down-regulated *VvIRT1* (*p* < 0.0001). The expression pattern of roots of intercropped 196.17 rootstocks grown at high Cu concentrations is equal to the one detected in roots of the monocropped 196.17 rootstock grown at the same Cu concentration (Figures 10A,E). Indeed the nutrient content was shown to be similar between mono- and intercropped 196.17 rootstocks: they are both characterized by high root Cu concentration (Figure 4), high Fe symplast content and low Mn apoplast concentration (Marastoni et al., 2019).

## Conclusion

In conclusion, our results demonstrate that the different adaptation of the two rootstocks to Cu toxicity is mainly achieved through a fine-tuning of morphological responses and bivalent cations transporter genes expression in the roots. Indeed, the enhanced root apparatus observed in Fercal rootstocks could be considered a mechanism to tolerate copper toxicity and the induced nutrient deficiency, since it allows plants to accumulate higher concentrations of metal ions in root apoplast and to explore a bigger soil volume, respectively. The 196.17 up-regulates the divalent cations transporters as a consequence of a possible Cu-induced Mn deficiency, whereas the Fercal rootstock does not respond to Cu toxicity at a molecular level. The intercropping between oat and grapevine seems to have a different effect depending on the rootstock and the Cu concentration in the nutrient solution. At low Cu concentrations (i.e., 0.2 and 5 μM Cu) both rootstocks up-regulate the transporters of bivalent cations, however, the 196.17 seems also to increase the apoplast affinity for divalent cations. Moreover, the intercropped 196.17 had a lower root length and surface area. Overall, these pieces of information suggest that, in a competing environment and without metal toxicity, Fercal rootstock explore the surrounding media (i.e., higher root surface), and induce the transporters of cations. Whereas, the 196.17 rootstock competes with the other species, increasing its ability to accumulate cations in the apoplast and inducing cation transporters. At 50 μM Cu, no differences between the root morphology of the two rootstocks were reported. However, at this Cu concentration Fercal induces the expression of *VvCtr2* suggesting that this rootstock does not use other strategies to manage Cu toxicity and Mn deficiency at the same time, whereas the 196.17 intercropped only up-regulate *VvNRAMP1* due to the Mn deficiency.

Overall, our results could impact the agronomic practice in viticulture, since the evidence provided with the present experiments show that the choice of specific rootstock genotype according with the soil conditions (e.g., heavy metal concentrations) might play an important role in the growth and the general health status of grapevine plants, that will eventually impact the productivity of the vineyard.

## Data Availability

The raw data supporting the conclusions of this manuscript will be made available by the authors, without undue reservation, to any qualified researcher.

## Author Contributions

LM, YP, SC, and TM designed the study. LM, MS, and FV performed the study. All authors analyzed the data, collected, and interpreted the data. LM, YP, FV, SC, and TM wrote the manuscript.

## Conflict of Interest Statement

The authors declare that the research was conducted in the absence of any commercial or financial relationships that could be construed as a potential conflict of interest. The handling Editor declared a past co-authorship with several of the authors YP, TM, and SC.

## Footnotes

1 http://www.vivairauscedo.com/portinnesti

2 http://genomes.cribi.unipd.it/grape/

## Supplementary Material

FIGURE S1 Representative root apparatus of the Fercal rootstocks at 25 μM Cu **(a)** showing the characteristic thickening of root tips caused by Cu toxicity **(b)**. A representative root tip of a Fercal rootstock grown without toxic Cu concentrations (i.e., 0.2 μM Cu) is also shown **(b)**.

FIGURE S2 Phylogenetic tree of *NRAMP* genes.

FIGURE S3 *VvCTr* genes expression determined in root apexes of monocropped Fercal **(a)** and 196.17 **(b)** rootstocks plants grown at 0.2 μM Cu. The data were normalized to two internal controls, the Elongation Factor 1α and the tubulin. The relative expression ratios were calculated using *VvCTr1* as calibrator. The values reported are means ± SE (*n* = 3).

TABLE S1 Sequences of primers used for the qRT-PCR.
