# Supplementary figures and images for: Morphological Root Responses and Molecular Regulation of Cation Transporters Are Differently Affected by Copper Toxicity and Cropping System Depending on the Grapevine Rootstock Genotype
Source: Front Plant Sci. 2019 Jul 19;10:946. doi: 10.3389/fpls.2019.00946 (PMC6658886; doi:10.3389/fpls.2019.00946)

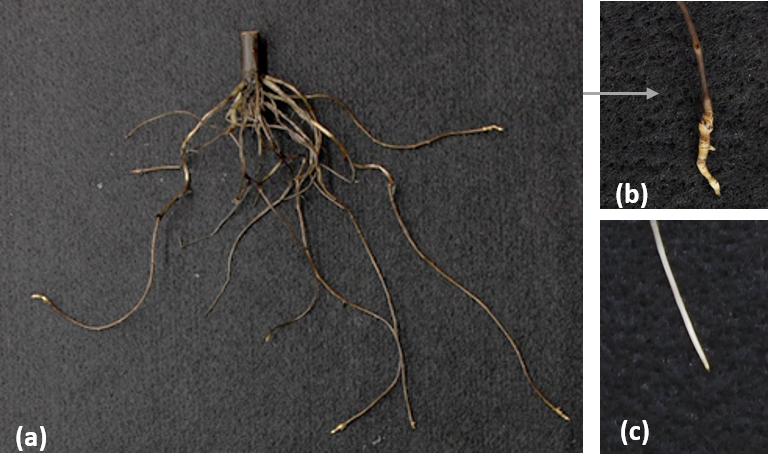

Supplement: FIGURE S1 — Representative root apparatus of the Fercal rootstocks at 25 μM Cu (a) showing the characteristic thickening of root tips caused by Cu toxicity (b). A representative root tip of a Fercal rootstock grown without toxic Cu concentrations (i.e., 0.2 μM Cu) is also shown (b). [file Image_1.TIF]

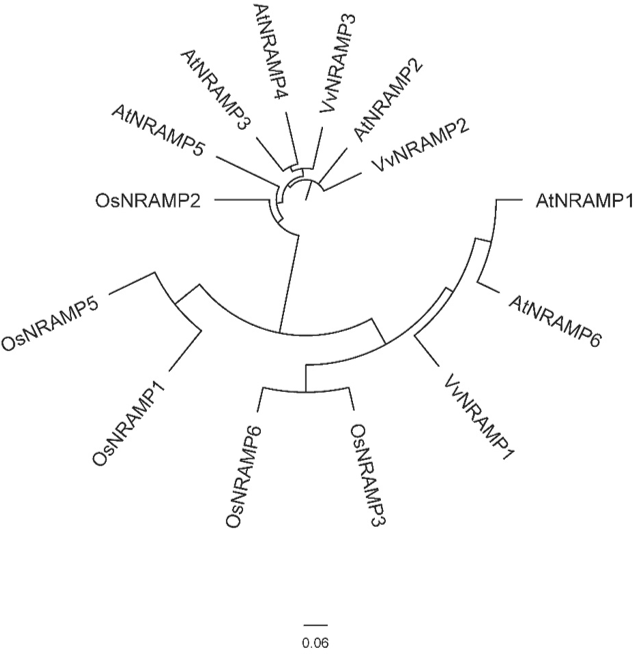

Supplement: FIGURE S2 — Phylogenetic tree of NRAMP genes. [file Image_2.TIF]

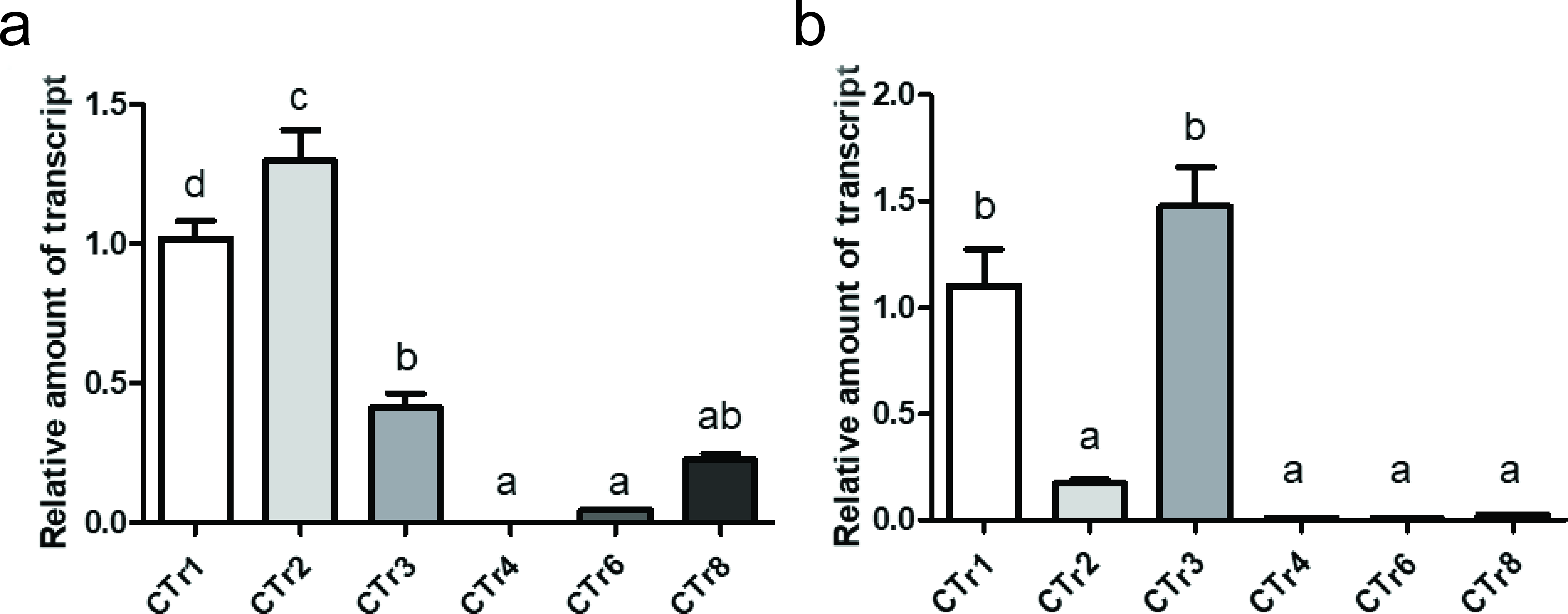

Supplement: FIGURE S3 — VvCTr genes expression determined in root apexes of monocropped Fercal (a) and 196.17 (b) rootstocks plants grown at 0.2 μM Cu. The data were normalized to two internal controls, the Elongation Factor 1α and the tubulin. The relative expression ratios were calculated using VvCTr1 as calibrator. The values reported are means ± SE (n = 3). [file Image_3.TIF]
