# Supplementary material for: Morphological Root Responses and Molecular Regulation of Cation Transporters Are Differently Affected by Copper Toxicity and Cropping System Depending on the Grapevine Rootstock Genotype
Source: Front Plant Sci. 2019 Jul 19;10:946. doi: 10.3389/fpls.2019.00946 (PMC6658886; doi:10.3389/fpls.2019.00946)
Supplement: TABLE S1 — Sequences of primers used for the qRT-PCR. [file Table_1.DOCX]

**Copper toxicity and intercropping trigger different responses and molecular regulation of cation transporters in grapevine rootstocks**

Laura Marastoni, Michele Sandri, Youry Pii*, Fabio Valentinuzzi, Stefano Cesco, Tanja Mimmo.

*Faculty of Science and Technology, Free University of Bozen-Bolzano, Piazza Università 5, I-39100 Bolzano, Italy*

**Table S1.** Sequence of primers used for the qRT-PCR.

| Gene | Primer | Ref |
| --- | --- | --- |
| *VvCtr1* | Fw:5’-CGGTTGGGTACTACTTGTTC-3’ | This work |
|  | Re: 5’-GAATCCAAACGGAGATCAAG-3’ |  |
| *VvCTr2* | Fw: 5’-TACATGCTCATGCTCGCCG-3’ |  |
|  | Re: 5’-GACCCGACTCCCGAAAATC-3’ |  |
| *VvCTr3e* | Fw: 5’-GGATTGGTCTGGCGTATATG-3’ |  |
|  | Re: 5’-AAACCCTACTCCCGAAAACC-3’ |  |
| *VvCTr4* | Fw: 5’-TGGAGTGGCTTTCCCATTTC-3’ |  |
|  | Re: 5’-CAGACCCATCCGAATAGCG-3’ |  |
| *VvCTr6* | Fw: 5’-cgtcttcgtcgtctctgtg-3’ |  |
|  | Re: 5’-TGAATAATGGTCTCGGCAGC-3’ |  |
| *VvCTr8* | Fw: 5’-AAGCGAAGGCGGTGTCTGG-3’ |  |
|  | Re: 5’-GCACTCCTCAGTCAATCCAC-3’ |  |
| *VvIRT1* | Fw: 5’-CGGAAATCGAAGTTGCAGAT-3’ | Vannozzi et al, 2017 |
|  | Re: 5’-GCTGCCACAAGAGGCTTTAT-3’ |  |
| *VvNRAMP1* | Fw: 5’-GAAGTCTTGCGTGGACTCTT-3’ | This work |
|  | Re: 5’-AGAGATTGTGCGGCATAACC-3’ |  |
| *VvNRAMP2* | Fw: 5’-CTGGGGTATTGGACTATTGG-3’ |  |
|  | Re: 5’-TTCTTCAGACGGAGATTGAG-3’ |  |
| *VvNRAMP3* | Fw: 5’-CATTGTGCCCACTATGATAG-3’ |  |
|  | Re: 5’-GGATCTGAATAGACTGAAGC-3’ |  |
| *EF1α* | Fw 5’-GTGCGTCATAGTTTTCTGCC-3’ | Pii et al., 2014 |
|  | Re 5’-AAAGAGGACACGACACAACAT-3’ |  |
| *Tubulin* | Fw 5’-CCCCTCTTCCTTTACTATGA-3’ |  |
|  | Re 3’ –TCTGGCACTATTACAACTGG-3’ |  |
